# Ultra-processed Food Intake and Long-Term Risk of Cardiovascular Disease Mortality among Patients with Myocardial Infarction: A Prospective Analysis of the Alpha Omega Cohort

**DOI:** 10.1016/j.cdnut.2026.109527

**Published:** 2026-09-01

**Authors:** Maria G Jacobo Cejudo, Daisy Coyle, Iris van Damme, Esther Cruijsen, Johanna M Geleijnse, Neha Khandpur

**Affiliations:** 1 Division of Human Nutrition and Health, Wageningen University, Wageningen, The Netherlands; 2 Food Policy Division, The George Institute for Global Health, Sydney, Australia; 3 Department of Nutrition, Harvard T.H. Chan School of Public Health, Boston, MA, United States; 4 Center for Epidemiological Research in Health and Nutrition, University of São Paulo, São Paulo, Brazil

**Keywords:** CVD mortality, CHD mortality, stroke mortality, patients, diet quality, ultra-processed diet, ultra-processed food, prospective analysis, cohort study, Netherlands

## Abstract

**Background:**

The impact of a diet high in ultra-processed foods (UPFs) on mortality risk in patients with cardiovascular disease (CVD) remains unclear.

**Objectives:**

We examined the diet quality of a high UPF diet and its association with risk of total CVD mortality, coronary heart disease (CHD) mortality, and stroke mortality in patients with prior myocardial infarction (MI).

**Methods:**

We analyzed data from 4365 patients with a history of MI in the Alpha Omega Cohort (79% males). Dietary intake at baseline was collected using a 203-item food-frequency questionnaire. Diet quality was assessed using the Dutch Healthy Diet Index adapted for patients with CVD (DHD-CVD, range 0–160). UPFs were identified and categorized according to the Nova classification system. Q1 and Q4 of %g/d of UPF represented a low and a high ultra-processed diet, respectively. Hazard ratios (HRs) for CVD, CHD, and stroke mortality were obtained from multivariable Cox regression models.

**Results:**

Mean UPF contribution to the diet was 18%g/d (Q1 compared with Q4 = 9%g/d compared with 29%g/d). Compared with Q1, patients in Q4 had higher intakes of total energy and lower intakes of fruit and vegetables. DHD-CVD scores decreased slightly across quartiles (Q1 compared with Q4 = 95 compared with 82). Over a median follow-up of 14 y, 1012 patients died from CVD (659 from CHD, 208 from stroke). Compared with Q1, a high ultra-processed diet was not consistently or significantly associated with CVD and CHD mortality. However, it was associated with a higher risk of stroke mortality (HR_per10% point increase_: 1.21; 95% confidence interval: 1.02, 1.42). This association was stronger in older patients, females, and those with a higher BMI, lower physical activity levels, and lower socioeconomic status and robust to sensitivity analysis.

**Conclusions:**

In patients withprior MI, a high UPF diet was lower in whole, cardioprotective foods. Our study highlights the adverse association of a high UPF diet with stroke mortality risk among patients who have previously experienced an MI.

## Introduction

Cardiovascular diseases (CVDs), principally coronary heart disease (CHD) and stroke, are the leading cause of global premature mortality and a major contributor to morbidity [1]. Between 1990 and 2019, global CVD prevalence doubled from 271 million to 523 million, and CVD mortality rose from 12.1 million to 18.6 million cases [1]. The economic burden of CVD is staggering. In 2021, the total cost of CVD in the European Union was estimated at €282 billion, of which 55% was incurred by health- and social care systems and 45% was incurred in non–health-care areas across unpaid care by family, lost productivity, work absence, and disability [2]. Although CVD mortality may have declined in the Netherlands, substantial morbidity persists [3,4]. Addressing known modifiable factors, like a healthy diet, in the management of CVD and to lower the likelihood of a secondary CVD event and CVD-related mortality, is therefore imperative [[5], [6], [7]].

An ultra-processed food (UPF)–dominant diet comprised primarily of UPF, industrial formulations of refined ingredients and cosmetic additives [8], is being investigated for its putative role in CVD pathology [9]. Meta-analyzed estimates from 22 prospective studies highlighted that high UPF intake compared with low intake can increase risk of CVD, coronary artery disease, and stroke by 17%, 23%, and 9% in healthy participants, respectively [10]. A high UPF diet shares many common characteristics with the established dietary risk factors for CVD including an underrepresentation of fruits, vegetables, legumes, whole grains, nuts and seeds, milk, fiber, calcium, omega-3 fatty acids from seafood, and PUFAs and an overrepresentation of red meat, processed meat, sugar-sweetened beverages, *trans*-fatty acids, and sodium [11,12]. Beyond the disequilibrated representation of food groups and an imbalance of nutrients, several processing-specific mechanisms of a high UPF diet may adversely influence CVD outcomes, including the physical food structure and the chemical composition like thepresence of cosmetic additives and neoformed compounds[13]. Biologically plausible pathways implicated in CVD etiology - altered glucose metabolism, gut microbiota composition, and serum lipid concentrations, obesity, inflammation, and oxidative stress - are also likely to be involved [14].

Despite growing evidence of the primary occurrence of CVD, we know very little about the role of a high UPF diet among patient populations, especially among patients with an existing CVD diagnosis. Patient groups would likely remain at high residual CVD risk despite receiving appropriate medical treatment. We hypothesize that any compromised vascular function and heightened inflammation associated with their existing condition may make them more vulnerable to the health harms of UPFs, despite risk factor management through cardiovascular treatment and drugs. Three Brazilian studies that examined UPF consumption in relation to coronary artery disease, peripheral artery disease, and stroke in patients with existing CVD found evidence of an adverse association. However, their findings were limited by their cross-sectional design, relatively small sample sizes, and lack of assessment of fatal CVD outcomes [[15], [16], [17]].

Our study conducted in a large cohort of MI survivors addresses these limitations by prospectively examining the association between UPF consumption and long-term CVD mortality, with its prospective design reducing concerns regarding reverse causation bias. The objectives of the present study were to: *1*) determine the nutritional quality of a high UPF diet; and *2*) assess the prospective association between a high UPF diet and CVD mortality. Informed by the existing evidence, we hypothesized that a high UPF diet would be associated with lower diet quality and higher risk of CVD-related mortality among patients with prior myocardial infarction (MI).

## Methods

### Study population

For this prospective study, we used data from the Alpha Omega Cohort (AOC) in the Netherlands, a prospective study of 4837 Dutch patients aged 60 to 80 y, with a verified history of MI ≤10 y before study enrollment [18]. Patients were recruited in collaboration with cardiologists from 32 Dutch hospitals between April 2002 and December 2006. Most of the patients were males (78%), who were on statins (86%) and antihypertensive medications (89%) at the time of enrollment. More details on the trial design have previously been published [18]. The study was approved by the Medical Ethics Committee of South-West Holland and by the local ethics committees of all participating hospitals.

For the current study, the inclusion of patients from 2002 to 2006 (trial recruitment phase) was considered as baseline. To ensure the validity of the analysis, we excluded patients if they had: *1*) missing data on total energy intake (*n =* 453); or *2*) implausibly high or low reported energy intakes (<800 or >8000 kcal/d for males, and <600 or >6000 kcal/d for females, *n =* 19); or *3*) missing data on CVD /CHD/stroke mortality (*n =* 0). The analytical sample consisted of a total of 4365 patients (Supplementary Figure 1).

### Assessment of the diet

Self-reported baseline dietary intake over the past month was captured using a 203-item food-frequency questionnaire (FFQ), which was an extended and adapted version of a validated questionnaire on fatty acid and cholesterol intake [19]. The Pearson correlation coefficient for energy intake was 0.83 for the FFQ compared with a dietary history measured over the same period [19]. Total energy and nutrient intakes were calculated from food consumption data using the 2006 Dutch Food Consumption Database [Nederlands Voedingsstoffenbestand (NEVO)] [20]. This FFQ has not been validated for UPF intake.

### Categorization of UPF

UPFs were assessed as part of the overall dietary intake. UPFs are the fourth group of the Nova categorization system that classifies food according to their degree and level of processing [8]. Nova group 1 is unprocessed or minimally processed food, e.g., grains, fruits, vegetables, legumes, nuts, tubers, milk, and meat. Nova group 2 are processed culinary ingredients, e.g., table salt, sugar, honey, vegetable oils, and butter. Nova group 3 includes processed food, e.g., canned vegetables in brine, salted or sugar-coated nuts, canned fish, freshly made breads, and cheeses. Nova group 4 is UPF, industrially manufactured food and drink that have undergone processes such as chemical modification, extrusion, fractioning, grinding, hydrogenation, hydrolysis, molding, or prefrying, e.g., carbonated soft drinks, sweet or savory packaged snacks, ice cream, confectionery, industrially processed breads and buns, breakfast “cereals,” margarine and other spreads, flavored yogurts, reconstituted meat products, and preprepared frozen or shelf-stable ready-to-eat/heat meals [8].

Two coauthors (IvD, EC) independently categorized the food and beverage items captured by the FFQ into 1 of the 4 Nova groups. The categorization process was in line with recommended best practices and was iterative, relied on definitions and examples of the Nova categories, and was informed by the year of dietary data collection, the sociodemographic characteristics of the sample, and the country context [21]. This conservative approach to categorization is likely to have misclassified multicomponent items as non-UPFs. Previously published UPF validation studies [22] suggest that the UPF exposure metrics generated using this approach underestimate true UPF intakes.

As a first step, a food list was created in which all variants of the original 203 FFQ items were captured as presented in the FFQ (e.g., sugar appeared in the food list as “Sugar in dairy products,” “Sugar in coffee,” “Sugar in cappuccino,” “Sugar in tea”), generating a total of 254 items for categorization. After the first round of categorization followed by discussion, a total of 214 of the 254 food items (84.2%) were assigned to a Nova group. Examples of items that remained uncategorized at this stage included “Semi-skimmed yogurt, custard, etc.”; “Unknown coffee milk”; “Other cold sauces”; “Liver- and kidney products”; “Savory pie, quiche”; “Skimmed yogurt, custard etc.” In the second iteration of categorization, 31 items were assigned to a Nova group, informed by the original question asked to the patients in the FFQ. For the third iteration, information on the specific NEVO item that contributed the most to the nutrition composition information of the FFQ item was considered in assigning a Nova group to the final 9 food items—8 of these were multicomponent items (“Savory pie, quiche”; “Hotchpot”; “Fiber-rich breakfast products,” “Other breakfast products,” “Onion soup,” “Tomato soup,” “Soup with legumes,” and “Other soup”), whereas 1 was a beverage (“Tap water or diet soda”). Their Nova group assignment for our main analysis was in a nonultra-processed group (i.e., Nova group 1 or 3). The proportion of items from each Nova group was as follows: Nova group 1 represented 35% of all items (90/254), Nova group 2 represented 6% (16/254), Nova group 3 represented 15% (37/254), and Nova group 4 represented 44% of all items (111/254). A complete list of the FFQ food items and their final Nova categorization can be found in Supplementary Table 1.

UPFs were categorized into subgroups that best represented the subset of items (Supplementary Table 2):

1. Breakfast cereals
2. Chocolate and confectionery
3. Distilled alcohol (excluded from the main analysis)
4. Ready-to-heat/eat meals
5. Regular or diet soda, sugary drinks
6. Savory snacks
7. Ultra-processed breads
8. Ultra-processed fats and oils for cooking
9. Ultra-processed grain-based snacks
10. Ultra-processed meats
11. Ultra-processed dairy-based desserts
12. Ultra-processed dairy-based drinks
13. Ultra-processed sauces and spreads

### Assessment of CVD mortality

This study focused on total CVD mortality as the primary endpoint, and mortality from CHD and stroke as secondary endpoints. The mortality status of patients was monitored through linkages with municipal registries, from baseline (2002–2006) through 31 December 2022. Follow-up for cause-specific mortality occurred at 3 instances. From 2002 to 2009, information was obtained from the national mortality registry [Statistics Netherlands (CBS)], treating physicians, and close family members. Primary and contributing causes of death were coded by an independent Endpoint Adjudication Committee, as described previously [18,23]. From 2010 to 2012, data on the primary and contributing causes of death were obtained from CBS. From 2013 to 2018, CBS provided data on the primary cause-of-death only, and treating physicians were asked to fill out an additional cause-of-death questionnaire (67% response rate), which was coded by study physicians. From 2019 onward, only data on primary causes of death were obtained from CBS. The endpoint CVD, CHD, or stroke was allocated to all patients for whom it was a primary or contributing cause-of-death, based on any of the data sources. Mortality coding was performed according to the International Classification of Diseases, 10th revision (ICD-10) [24]. CVD mortality comprised I20–I25 (ischaemic heart disease), I46 (cardiac arrest), R96 (sudden death, undefined), I50 (heart failure), and I60–I69 (stroke). CHD mortality comprised ICD-10 codes I20–I25, I46, and R96. Stroke mortality comprised codes I60–I69.

### Assessment of covariates

At baseline, data were collected on demographic and anthropometric factors, lifestyle, current health status, and medical history. This included self-reported age, sex, education attainment, and smoking. Physical activity was assessed using the validated Physical Activity Scale of the Elderly [25]. Socioeconomic status was assessed using an area-based socioeconomic status indicator on zip code level from CBS [26]. This indicator includes data from the percentile of the welfare of the households in the area, educational level of the households, and recent labor activities of the households. A higher score indicates a higher socioeconomic status (SES- WOA). Self-rated health was assessed with the question “how do you rate your overall health at this moment?” using a 5-point scale ranging from poor to excellent. Marital status was assessed with the question “what is your marital status?” with 6 possible responses. Self-reported medication use was checked by research nurses and coded according to the Anatomical Therapeutic Chemical classification system [27]. Total alcohol intake was estimated from FFQ data by summing the ethanol content of all alcoholic beverages consumed. Serum lipids were analyzed from nonfasting blood using standard kits and an automated analyzer (Hitachi 912; Roche Diagnostics). Blood pressure was measured twice using an automatic device and averaged (HEM-711; Omron). BMI was calculated from the measured weight and height in kg/m^2^.

### Statistical analysis

#### Operationalization of the ultra-processed diet indicator

For each participant, we calculated the total grams of UPF relative to the total grams of food and beverages consumed per day (% g of UPF/d) to represent the ultra-processed diet indicator. Quartiles of % grams of UPF per day were chosen as the primary exposure variable, as published evidence suggests that weight ratio may be a better indicator of UPF intake than relative energy contribution [22], while capturing several dimensions of the food that do not provide energy (e.g., additives) and accounting for several food items with low- or no calories (e.g., diet soda). Quartiles 1 and 4 of % g/d of UPF represented a low and a high ultra-processed diet, respectively. Continuous exposures of per 10%-point increments of UPF intake (% g/d) and per 1 SD increment in energy-adjusted UPF intake (corresponding to ∼170 g/d) using the residual method [28] were used to assess dose–response. For energy adjustment, we calculated standardized residuals by regressing the consumption of UPF (g/d) on total energy intake. Alcohol intake was excluded from all Nova group indicators. Total alcohol intake (g/d) that captured the consumption of both fermented and distilled alcohol including “Mixed alcoholic drinks,” “Strong liquor,” “All wine,” “Sherry, vermouth and port,” “Low-alcohol beer,” “Beer,” and “Other alcoholic beverages” was adjusted for in the analytical models as a confounder. This was done to avoid conflating alcohol-related effects with dietary processing, because alcohol consumption has been shown to have a strong, independent, and nonlinear influence on CVD mortality in this population [29].

#### Operationalization of diet quality

The diet quality across quartiles of UPF intake was captured by the content of key nutrients and adherence to the Dutch dietary guidelines for patients with CVD (DHD-CVD) [30]. This index assesses adherence to the current Dutch dietary guidelines for patients with atherosclerotic CVD with special consideration to the intake of fish and plant sterol or stanol-enriched products [31,32] and assigns scores ranging from 0 to 160 for adherence to 16 food groups. Mean or median intakes of all the different Nova groups, UPF subgroups, dietary macronutrients—total carbohydrates, total fiber, free sugars (includes added and naturally occurring sugars in honey and syrups), protein, total fat, saturated fat, monounsaturated fat, polyunsaturated fat, total alcohol—and micronutrients including key minerals and vitamins, were estimated. The nonbeverage energy density (kcal/g from solids) and the nonalcoholic beverage energy density (kcal/mL from liquids, excluding alcohol) across UPF quartiles were also estimated.

#### Operationalization of the CVD mortality indicator

The primary outcome was a binary indicator capturing total CVD-related mortality during long-term follow-up (from baseline through 2022, mean follow-up time ∼14 y), whereas secondary outcomes were binary indicators for CHD and stroke-related mortality.

#### Operationalization of the covariates

Educational attainment was classified into 4 categories: only elementary, low, moderate, and high. Smoking status comprised 3 categories: never, former, or current smoker. Physical activity was categorized as low (no activity or only light activity; <3 metabolic equivalent tasks (METs)], intermediate [moderate and/or vigorous leisure activity (*≥*3 METs) on *>*0 to *<*5 d/wk], and high [moderate or vigorous activity (*≥*3METs) on ≥5 d/wk]. Marital status was a categorical variable with 6 options: unmarried, cohabiting, married, widow(er), divorced, or unwilling to answer. Alcohol (total ethanol) intake in males was categorized as 0 to 2 g/d (none to very light), *>*2 to 10 g/d (light), *>*10 to 30 g/d (moderate), and*>*30 g/d (heavy). In females, the categories were 0 to 2 g/d (none-very light), *>*2 to 5 g/d (light), *>*5 to 15 g/d (moderate), and *>*15 g/d (heavy). BMI ≥ 30 kg/m^2^ was classified as obese.

#### Statistical tests

All analyses were conducted in R version 4.0.2 (R Foundation for Statistical Computing) [33]. A 2-sided *P* value of < 0.05 was considered statistically significant. Descriptive analyses were performed for diet quality. We estimated person-years from the age of the participant at the time of study enrollment to the age at date of CVD, CHD, or stroke mortality, or the end of the follow-up (31 December, 2022), whichever came first.

We used Cox proportional hazards regression models to calculate hazard ratios (HRs) and 95% confidence intervals (CIs) for CVD, CHD, or stroke mortality. Age was used as the underlying time scale in all Cox models. Entry time was defined as age at study enrollment (delayed entry), thereby accounting for left truncation, and exit time was either age at diagnosis of death from CVD, death from other causes, loss to follow-up, or end of follow-up, whichever occurred first. Fifteen patients were lost to follow-up and censored. A stepwise/forward modeling approach was considered. Model 1 was adjusted for sex. Model 2 was additionally adjusted for the following socioeconomic and lifestyle factors: educational attainment, SES-WOA, smoking, physical activity level, and total ethanol intake. Model 3 was additionally adjusted for total energy intake (kcal/d). Medication use was considered but not included in the models as almost all patients were on similar medication regimens. For all covariates except SES-WOA (14.5% missing), ≤1% of values were missing. To avoid extensive imputation for a nonnegligible number of patients or exclusion of those with missing data and risk of selection bias, we imputed missing data using age and sex-specific modal values (for categorical variables) or median (for continuous variables) in the main analysis. This was the parsimonious choice to avoid introducing additional modeling assumptions. The proportionality of the hazards’ assumption was tested with Schoenfeld residuals, and it was met for all the outcomes, except for smoking, for which a strata function was incorporated. The linearity assumption was visually checked using martingale residuals, and it was met for all outcomes [34].

We ran several sensitivity analyses. First, we additionally adjusted for BMI in the main analysis (model 3). Second, we repeated our main analysis using an alternate UPF metric of quartiles of %kcal of UPF per day. Third, we repeated the main analysis using sex-specific UPF quartiles, as females tend to eat healthier than males. Fourth, we repeated the main analysis by excluding patients who died from CVD, CHD, and stroke during the first 3 y of follow-up to account for possible changes in diet preceding mortality in this group. Fifth, we repeated the main analyses by adjusting for energy from Nova groups 1, 2, and 3, instead of total energy to test if energy from UPF influenced the outcomes of the study. Finally, we additionally adjusted for diet quality in model 3. Seventh, we tested interactions using the main model (model 3) and stratified by age (younger and older based on the median), sex, diet quality (low and high based on the median DHD-CVD score), BMI (obese compared with nonobese), physical activity (no-light and moderate-high), and socioeconomic status (low and high based on the median SES-WOA score). Eighth, we also repeated the main analysis by imputing missing data on the following covariates using multiple imputation with chained equations (10 imputed datasets, 10 iterations) using the Multivariate Imputation by Chained Equations (MICE) package: smoking (*n =* 1), BMI (*n =* 6), physical activity (*n =* 25), educational attainment (*n =* 24), and SES-WOA (*n =* 634). The analyses were performed for each imputed dataset separately, and the estimates were subsequently combined using Rubin’s rules. Ninth, we additionally adjusted for sodium intake from food. Finally, we checked if the intake of minimally processed food (in %g/d) showed a reverse association with the study outcomes to that seen with UPF.

## Results

A majority of the study sample was male, married, former smokers, with light-to-moderate alcohol intake. The mean BMI was 27.7 kg/m^2^ and most patients had intermediate-high physical activity levels and considered themselves to be in good health (Table 1). Baseline sociodemographic characteristics, lifestyle behaviors, and anthropometric measurements of study patients were similar across quartiles of ultra-processed diets. However, compared with patients in Q1, a larger proportion of patients in Q4 were males (87% compared with 65%), married, with lower education attainment, lower activity levels, current smokers, and without prevalent diabetes.

**TABLE 1:** Baseline characteristics of 4365 post-myocardial infarction patients of the Alpha Omega Cohort, overall and across quartiles percentage UPF consumption^a^

		Quartiles of UPF intake (%g/d)			
	Total	Q1	Q2	Q3	Q4
	*n* = 4365	*n* = 1092	*n* = 1091	*n* = 1090	*n* = 1092
Age (y), median (IQR)	68.7 (64.3–73.4)	69.0 (64.7–73.7)	69.0 (64.6–73.4)	68.7 (64.1–73.5)	68.2 (63.8–72.8)
Females, *n* (%)	933 (21)	387 (35)	260 (24)	147 (14)	139 (13)
Dutch-origin parents, *n* (%)	4311 (98.8)	1072 (98.2)	1080 (99.0)	1082 (99.3)	1077 (98.6)
Years since last MI, median (IQR)	3.7 (1.7–6.3)	3.6 (1.7–6.3)	3.8 (1.7–6.3)	3.8 (1.7–6.4)	3.6 (1.6–6.4)
Marital status, *n* (%)					
Unmarried	144 (3.3)	36 (3.3)	37 (3.4)	32 (2.9)	39 (3.6)
Cohabiting	116 (2.7)	34 (3.1)	23 (2.1)	27 (2.5)	32 (2.9)
Married	3474 (79.7)	844 (77.4)	856 (78.5)	897 (82.5)	877 (80.4)
Widow(er)	464 (10.6)	123 (11.3)	138 (12.7)	91 (8.4)	112 (10.3)
Divorced	154 (3.5)	51 (4.7)	35 (3.2)	40 (3.7)	28 (2.6)
SES-WOA, median (IQR)	0.06 (–0.12, 0.18)	0.05 (–0.14, 0.19)	0.06 (–0.09, 0.18)	0.07 (–0.09, 0.18)	0.05 (–0.14, 0.18)
Educational attainment, *n* (%)					
Only elementary	882 (20)	185 (17)	204 (19)	233 (22)	260 (24)
Low	1557 (36)	386 (35)	340 (31)	403 (37)	428 (39)
Moderate	1367 (32)	355 (33)	383 (35)	327 (30)	302 (28)
High	535 (12)	159 (15)	159 (15)	120 (11)	97 (9)
Smoking, *n* (%)					
Never	722 (17)	242 (22)	194 (18)	146 (14)	140 (13)
Former	2929 (67)	703 (65)	713 (65)	766 (70)	747 (68)
Current	713 (16)	146 (13)	184 (17)	178 (16)	205 (19)
Alcohol intake, *n* (%)					
No or very light	1336 (30)	378 (35)	314 (29)	300 (27)	344 (31)
Light	1125 (26)	247 (23)	278 (25)	289 (27)	311 (29)
Moderate	1212 (28)	279 (25)	306 (28)	339 (31)	288 (26)
Heavy	692 (16)	188 (17)	193 (18)	162 (15)	149 (14)
Physical activity, *n* (%)^b^					
No or light activity	1783 (41)	438 (41)	411 (38)	466 (43)	468 (43)
Intermediate	923 (21)	221 (20)	244 (23)	218 (20)	240 (22)
High	1634 (38)	423 (39)	427 (39)	402 (37)	382 (35)
Waist circumference (cm), mean ± SD	102 ± 10	101 ± 11	101 ± 10	103 ± 10	103 ± 10
BMI (kg/m^2^), mean ± SD	27.7 ± 3.8	27.9 ± 3.9	27.6 ± 3.7	27.7 ± 3.6	27.7 ± 3.9
BMI categories, *n* (%)					
Normal	990 (23)	234 (22)	278 (26)	229 (21)	249 (23)
Overweight	2337 (54)	579 (53)	550 (50)	629 (58)	579 (53)
Obese	1032 (24)	276 (25)	263 (24)	232 (21)	261 (24)
Self-rated health, *n* (%)					
Very good–excellent	510 (12)	107 (10)	157 (14)	126 (12)	120 (11)
Good	2827 (65)	709 (65)	696 (64)	730 (67)	692 (64)
Poor–moderate	1010 (23)	273 (25)	233 (22)	231 (21)	273 (25)
Medication use, *n* (%)					
Insulins and analogs	237 (5)	67 (6)	60 (5)	61 (6)	49 (5)
Oral glucose-lowering drugs	505 (12)	146 (13)	113 (10)	125 (12)	121 (11)
Metformin	352 (8)	93 (9)	80 (7)	87 (8)	92 (8)
Antihypertensives	3919 (90)	983 (90)	984 (90)	962 (88)	990 (91)
Statins	3747 (86)	925 (85)	951 (87)	945 (87)	926 (85)
Prevalent diabetes, *n* (%)	883 (20)	258 (24)	197 (18)	220 (20)	208 (19)
Blood pressure (mm Hg), mean ± SD					
Systolic pressure	142 ± 22	143 ± 22	142 ± 21	141 ± 21	141 ± 21
Diastolic pressure	80 ± 11	80 ± 11	80 ± 11	80 ± 11	80 ± 11
Serum lipids, mmol/L, mean ± SD					
Total cholesterol	4.7 ± 1.0	4.8 ± 1.0	4.7 ± 1.0	4.7 ± 0.9	4.7 ± 0.9
LDL cholesterol	2.6 ± 0.8	2.6 ± 0.8	2.5 ± 0.8	2.6 ± 0.8	2.6 ± 0.8
HDL cholesterol	1.3 ± 0.3	1.3 ± 0.4	1.3 ± 0.4	1.3 ± 0.3	1.2 ± 0.3
Triglycerides	1.9 ± 1.1	1.9 ± 1.1	1.9 ± 1.0	1.9 ± 1.1	1.9 ± 1.0
In intervention arm during RCT, *n* (%)	3258 (75)	830 (76)	814 (75)	819 (75)	795 (73)

Abbreviations: MET, metabolic equivalent of task; MI, myocardial infarction; RCT, randomized controlled trial; SES-WOA, socioeconomic status-WOA (socioeconomic status—wealth, education, and occupation); UPF, ultra-processed food.

a All values are mean ± SD for normally distributed continuous data, median (IQR) for skewed continuous data, and *n* (%) for categorical data. Missing values per covariable: *n =* 38 for years since last MI; *n =* 6 for marital status (+7 unwilling to answer), BMI, systolic and diastolic blood pressure; *n =* 2 for Dutch-origin parents; *n =* 25 for waist circumference and physical activity; *n* = 24 for educational attainment; *n* = 1 for basal smoking and antidiabetic medication; *n =* 18 for self-rated health; *n =* 111 for total cholesterol, HDL cholesterol, and triglycerides; and *n =* 309 for LDL cholesterol.

b Physical activity was categorized as low (no activity or only light activity, ≤3 METs), intermediate (>0 to <5 d/wk of moderate or vigorous activity, >3 METs), or high (≥5 d/wk of moderate or vigorous activity, >3 METs).

Mean total energy intake was 1914 ± 521 kcal (Q1= 1703 kcal; Q4 = 2041 kcal). The mean absolute contribution from UPF was 962 ± 361 kcal, corresponding to 53 ± 12% of total kcals consumed (Q1: 42%; Q4: 62%). Mean UPF intake was 415 ± 204 g/d corresponding to 18% ± 8% of total weight of food and drinks consumed (Q1: 9%; Q4: 29%). The higher contribution of UPF to total energy intake compared with weight (i.e., 53% kcal compared with 18% g) is expected given their energy density relative to minimally processed foods, a discrepancy that has been consistently reported in previous studies.

The diet quality score was higher in Q1 than in Q4, but with a narrow range (DHD-CVD median scores for Q1 compared with Q4 = 95 compared with 82, Table 2). Compared with the lower quartiles, the diet of patients in the highest quartiles was characterized by higher intakes of total energy, more frequent meal skipping, and lower intakes of processed food and unprocessed or minimally processed food, particularly fruit. Compared with those in Q1, Q4 patients had especially high intakes of the following UPF subgroups: confectionery, ready-to-eat meals, ultra-processed bread, ultra-processed grain-based snacks/desserts, sauces and spreads, and sugar-sweetened beverages. Intake of total and saturated fat, carbohydrates, free sugars, and sodium was only slightly higher in Q4, whereas intake of potassium was lower (Table 2).

**TABLE 2:** Dietary characteristics of 4365 post-myocardial infarction patients of the Alpha Omega Cohort, overall and across quartiles percentage UPF consumption^a^

		Quartiles of UPF intake, g/d			
	Total	Q1 ≤12%	Q2 >12–≤16%	Q3 >16–≤22%	Q4 >22%
	*n =* 4365	*n =* 1092	*n =* 1091	*n =* 1090	*n =* 1092
Total energy intake (kcal/d) (excluding alcohol)	1801 ± 500	1596 ± 447	1765 ± 450	1916 ± 493	1928 ± 531
Total energy intake (kcal/d) (including alcohol)	1914 ± 521	1703 ± 474	1883 ± 471	2031 ± 514	2041 ± 550
UPF intake (g/d)	415 ± 204	241 ± 77	351 ± 89	446 ± 118	622 ± 245
UPF intake (kcal/d)	962 ± 361	664 ± 229	906 ± 260	1080 ± 311	1199 ± 378
% grams/day UPF	18 ± 8	9 ± 2	14 ± 1	19 ± 2	29 ± 7
% kcal/day UPF	53 ± 12	42 ± 10	52 ± 8	56 ± 8	62 ± 9
Energy density of the overall solid diet (kcal/g)	1.6 ± 0.3	1.4 ± 0.3	1.6 ± 0.3	1.7 ± 0.3	1.7 ± 0.3
Energy density of solid UPF (kcal/g)	2.9 ± 0.5	3.0 ± 0.6	2.9 ± 0.5	2.9 ± 0.5	2.8 ± 0.5
Energy density of beverages (including alcohol) (kcal/mL)	0.2 (0.1–0.3)	0.2 (0.1–0.2)	0.2 (0.1–0.2)	0.2 (0.1–0.3)	0.2 (0.2–0.3)
Energy density of UP beverages (excluding alcohol) (kcal/mL)	0.4 (0.0–0.6)	0.0 (0.0–0.4)	0.4 (0.0–0.6)	0.4 (0.3–0.7)	0.4 (0.4–0.6)
Frequency of meal consumption, *n* (%)^2^					
Breakfast					
No or < 4 d/wk	193 (4)	46 (4)	40 (4)	43 (4)	64 (6)
Between 4 and 6 d/wk	559 (13)	143 (13)	131 (12)	135 (12)	150 (14)
Every day or >6 d/wk	3569 (82)	895 (82)	911 (84)	904 (83)	859 (79)
Lunch					
No or < 4 d/wk	225 (5)	64 (6)	49 (5)	55 (5)	57 (5)
Between 4 and 6 d/wk	630 (14)	164 (15)	137 (13)	158 (15)	171 (16)
Every day	3444 (79)	850 (78)	886 (81)	863 (79)	845 (77)
Dinner					
No or < 4 d/wk	79 (2)	23 (2)	14 (1)	13 (1)	29 (3)
Between 4 and 6 d/wk	839 (19)	201 (18)	194 (18)	198 (18)	246 (23)
Every day	3438 (79)	865 (79)	882 (81)	878 (81)	813 (75)
Nova 1–3 food groups intake					
Unprocessed or minimally processed food (g/d)	1851 ± 598	2249 ± 621	1969 ± 494	1768 ± 458	1418 ± 474
Unprocessed or minimally processed food (kcal/d)	539 ± 188	595 ± 212	554 ± 178	541 ± 170	466 ± 163
Processed culinary ingredients (g/d)	7 (1–23)	5 (1–18)	7 (1–25)	8 (1–27)	7 (1–23)
Processed culinary ingredients (kcal/d)	37 (7–119)	31 (6–96)	37 (7–123)	45 (8–131)	40 (6–120)
Processed food (g/d)	120 (65–200)	149 (82–243)	126 (73–203)	114 (61–183)	96 (52–171)
Processed food (kcal/d)	184 (102–297)	229 (129–365)	194 (114–295)	163 (97–275)	150 (83–251)
Nova group 1 subgroups intake (g/d)					
Beans, legumes, nuts	16 ± 11	16 ± 11	16 ± 11	17 ± 11	16 ± 12
Fruits	110 (43–251)	160 (86–296)	116 (63–263)	108 (43–223)	85 (21–123)
Leafy-cruciferous veggies	39 ± 22	41 ± 22	41 ± 22	39 ± 21	36 ± 21
Other vegetables	26 ± 18	28 ± 18	27 ± 18	26 ± 17	23 ± 17
Meat-based dishes	32 (16–46)	28 (13–45)	32 (17–46)	34 (17–49)	31 (14–46)
Roots and tubers	103 ± 48	100 ± 50	105 ± 47	108 ± 46	98 ± 48
Nova group 4 subgroups intake (g/d)					
Confectionery	20 (9–36)	15 (5–27)	20 (10–32)	23 (12–39)	23 (11–42)
Ready-to-eat/heat meals	28 (11–57)	18 (7–36)	28 (12–51)	35 (15–69)	35 (14–79)
Savory snacks	10 (3–18)	5 (1–13)	9 (3–18)	12 (4–20)	12 (5–21)
Ultra-processed breads	124 ± 62	83 ± 52	121 ± 55	143 ± 56	149 ± 60
Fats and oils	7 (0.4–21)	4 (0–16)	7 (1–21)	9 (1–24)	8 (0–25)
Sauces and spreads	30 ± 22	22 ± 16	28 ± 19	33 ± 24	35 ± 24
Ultra-processed grain-based snacks/desserts	23 (12–44)	17 (11–36)	25 (13–45)	26 (16–46)	26 (12–47)
Ultra-processed meat	17 (7–40)	13 (6–19)	17 (7–35)	17 (7–42)	18 (8–44)
Dairy-based desserts	13 (0–24)	11 (0–15)	13 (0–21)	15 (0–34)	15 (0–36)
Sweet milk	0 (0–21)	0 (0–0)	0 (0–21)	0 (0–25)	0 (0–48)
Sugar-sweetened beverages	21 (0–63)	0 (0–21)	0 (0–42)	21 (0–75)	107 (21–213)
Macronutrient intake (g/d)					
Total protein (g/d)	70 ± 19	67 ± 19	70 ± 18	73 ± 19	71 ± 20
Vegetable protein	27 ± 8	205 ± 7	27 ± 8	29 ± 8	28 ± 8
Animal protein	43 ± 15	43 ± 16	43 ± 15	45 ± 15	43 ± 16
Total fatty acids (g/d)	68 ± 25	56 ± 21	66 ± 22	75 ± 25	76 ± 28
SFAs	27 ± 11	22 ± 9	26 ± 10	29 ± 11	30 ± 12
MUFAs	24 ± 9	20 ± 8	23 ± 8	26 ± 9	26 ± 10
PUFAs	16 ± 8	13 ± 6	15 ± 7	17 ± 8	18 ± 8
*Trans*-fatty acids	1.6 ± 0.6	1.3 ± 0.5	1.5 ± 0.6	1.7 ± 0.6	1.7 ± 0.7
Cholesterol (mg/d)	182 ± 69	160 ± 61	179 ± 61	194 ± 73	194 ± 76
Total carbohydrates (g/d)	223 ± 68	203 ± 70	219 ± 64	233 ± 67	237 ± 70
Total monosaccharides and disaccharides	116 ± 50	110 ± 49	112 ± 47	118 ± 49	123 ± 54
Total dietary fiber	21 ± 7	21 ± 7	22 ± 7	22 ± 7	21 ± 7
Free sugar	101 ± 47	94 ± 46	98 ± 44	103 ± 46	109 ± 50
Total added sugar to coffee, tea, and dairy	0 (0–16)	0 (0–9)	0 (0–16)	2 (0–18)	1 (0–17)
Total alcoholic beverages (mL/d)	81 (15–193)	66 (14–168)	81 (17–190)	93 (24–200)	87 (15–201)
Total ethanol (g/d)	7 (1–17)	6 (1–16)	8 (2–19)	8 (2–17)	6 (1–17)
Micronutrients (mg/d)					
Sodium	2,219 ± 675	1,941 ± 583	2,185 ± 618	2,364 ± 669	2,386 ± 728
Potassium	3,251 ± 861	3,359 ± 908	3,297 ± 823	3,319 ± 848	3,028 ± 823
Calcium	906 ± 382	947 ± 425	918 ± 365	904 ± 362	855 ± 367
Iron	10.0 ± 3.0	10.0 ± 2.5	10.5 ± 2.7	10.9 ± 2.8	10.3 ± 2.8
Magnesium	302 ± 78	302 ± 80	306 ± 76	310 ± 77	292 ± 77
Vitamins					
Retinol (μg)	576 (384–923)	436 (303–670)	576 (392–898)	641 (434–1016)	677 (442–1057)
Beta-carotene (μg)	1676 ± 855	1685 ± 881	1702 ± 869	1732 ± 872	1,586 ± 788
Alpha-carotene (μg)	231 (130–334)	228 (128–338)	234 (130–336)	245 (151–339)	214 (117–321)
Vitamin C (mg)	97 ± 54	112 ± 60	101 ± 53	94 ± 52	80 ± 45
Vitamin E (mg)	12.0 ± 5	10.3 ± 4.3	12.0 ± 5.0	13.2 ± 5.9	12.8 ± 5.9
Diet quality					
DHD-index for patients with CVD (0–160)	89 ± 15	95 ± 14	91 ± 14	88 ± 13	82 ± 14

Abbreviations: CVD, cardiovascular disease; DHD, Dutch Healthy Diet Index; UPF, ultra-processed food.

a All values are mean ± SD for normally distributed continuous data, median (IQR) for skewed continuous data, and *n* (%) for categorical data. Missing values: 44 for breakfast frequency; 66 for lunch frequency; 9 for dinner frequency.

Over a median follow-up time of 14 calendar years and 56,065 person-years, 1012 patients died from CVD, of which 659 died from CHD and 208 from stroke. After adjusting for confounders (model 3), a high UPF diet (Q4) showed no significant association with risk of mortality from total CVD compared with a low UPF diet (Q1). Among CVD subtypes, no association was seen for CHD mortality. A nonsignificant association was found for a high compared with a low UPF diet and higher stroke mortality risk (HR_Q4 compared with Q1_: 1.47; 95% CI: 0.98, 2.22) (Table 3). Dose–response analyses also showed a nonsignificant association with CVD and CHD mortality risk but a significant association with stroke mortality risk (HR: 1.21; 95% CI: 1.02, 1.42) for a continuous increase of 10% points in UPF intake (% g/d). Results per 1SD increase in energy-adjusted UPF intake (g/d) also showed significant associations with the risk of stroke mortality. CVD and CHD mortality tended toward a nonsignificant increase in HRs (Table 3).

**TABLE 3:** Hazard ratios (95% CIs) between UPF consumption and risk of mortality from CVD, CHD, and stroke, among 4365 post-myocardial infarction patients of the Alpha Omega Cohort^a^

	UPF quartiles (% g/d)				Per 10% point increment (% g/d)	Per 1 SD increment in energy-adjusted UPF intake (169 g/d)
	Q1 ≤12%	Q2 >12–≤16%	Q3 >16–≤22%	Q4 >22%		
Total CVD mortality						
Noncases/cases	811/281	816/275	813/277	813/279	3253/1112	3253/1112
Person-years	13,977	13,880	14,192	14,006	56,055	56,055
Crude model	Ref.	1.01 (0.85, 1.19)	1.02 (0.87, 1.21)	1.11 (0.94, 1.31)	1.07 (0.99, 1.15)	1.08 (1.02, 1.15)∗
Model 1	Ref.	1.01 (0.85, 1.19)	1.03 (0.87, 1.22)	1.12 (0.94, 1.32)	1.07 (1.00, 1.16)	1.08 (1.02, 1.15)∗
Model 2	Ref.	0.99 (0.84, 1.17)	0.99 (0.84, 1.18)	1.04 (0.88, 1.24)	1.04 (0.97, 1.12)	1.06 (1.00, 1.13)
Model 3	Ref.	0.99 (0.83, 1.17)	0.99 (0.83, 1.17)	1.04 (0.87,1.24)	1.04 (0.96, 1.12)	1.06 (1.00, 1.13)
Total CHD mortality						
Noncases/cases	915/177	928/163	937/153	926/166	3706/659	3706/659
Person-years	13,974	13,878	14,190	14,007	56,049	56,049
Crude model	Ref.	0.94 (0.76, 1.17)	0.89 (0.71, 1.10)	1.02 (0.83, 1.27)	1.04 (0.95, 1.15)	1.06 (0.99, 1.15)
Model 1	Ref.	0.93 (0.75, 1.15)	0.86 (0.69, 1.07)	0.99 (0.80, 1.23)	1.03 (0.94, 1.14)	1.06 (0.98, 1.15)
Model 2	Ref.	0.91 (0.74, 1.13)	0.83 (0.67, 1.04)	0.93 (0.75, 1.16)	1.00 (0.91, 1.11)	1.05 (0.97, 1.13)
Model 3	Ref.	0.92 (0.74, 1.14)	0.84 (0.67, 1.05)	0.94 (0.75, 1.18)	1.01 (0.91, 1.12)	1.05 (0.97, 1.14)
Total stroke mortality						
Noncases/cases	1046/46	1046/45	1032/58	1033/59	4157/208	4157/208
Person-years	13,972	13,876	14,188	14,001	56,037	56,037
Crude model	Ref.	1.01 (0.67, 1.52)	1.32 (0.89, 1.94)	1.44 (0.97, 2.12)	1.19 (1.02, 1.39)∗	1.21 (1.07, 1.36)∗
Model 1	Ref.	1.03 (0.68, 1.56)	1.38 (0.93, 2.05)	1.51 (1.01, 2.24)∗	1.21 (1.03, 1.42)∗	1.21 (1.07, 1.36)∗
Model 2	Ref.	0.98 (0.65, 1.48)	1.37 (0.92, 2.04)	1.47 (0.99, 2.20)	1.21 (1.03, 1.42)∗	1.21 (1.08, 1.37)∗
Model 3	Ref.	0.98 (0.64, 1.48)	1.37 (0.91, 2.06)	1.47 (0.98, 2.22)	1.21 (1.02, 1.42)∗	1.21 (1.08, 1.37)∗

Abbreviations: CI confidence interval; CVD, cardiovascular disease; CHD, coronary heart disease; SES-WOA, socioeconomic status-WOA (socioeconomic status—wealth, education, and occupation); UPF, ultra-processed food.

a Model 1 was adjusted for sex. Model 2 was additionally adjusted for educational attainment (categories), SES-WOA (continuous), smoking (categories), physical activity (categories), and total ethanol intake (categories). Model 3 was additionally adjusted for total energy intake (kcal/d). ∗ p-value <= 0.05

The main results were largely corroborated by sensitivity analysis. A high compared with low UPF diet showed a significant increase in hazards for stroke mortality across sex-specific quartiles, and when patients with CVD mortality in the first 3 y were excluded (Supplementary Table 3). Associations for stroke mortality risk remained positive and significant when continuous increases in UPF intake were modeled across all sensitivity tests. No associations were seen for total CVD and CHD mortality risk (Supplementary Table 3). Main results also remained robust across stratified analysis (Supplementary Tables 4–6). A high UPF diet tended toward higher risk for stroke mortality among older patients, females, those with a higher BMI, a higher diet quality, lower physical activity, being in a lower socioeconomic stratum, former smokers, and those who were assigned to the intervention group in the Alpha Omega Trial (Supplementary Table 6). However, the only significant interaction was observed between UPF intake and the DHD-CVD score for CHD outcome (*P*-interaction = 0.02). Finally, as hypothesized, a higher unprocessed and minimally processed diet had a protective association on the HR of total CVD and stroke mortality risk (Supplementary Table 7).

## Discussion

This study assessed the relative nutritional quality of a high UPF diet and the prospective association between a high UPF diet and CVD mortality in patients with history of MI. The difference in UPF intakes between the lowest and the highest UPF consumers was considerable in this sample of patients. We found limited evidence of a difference in the relative nutritional quality of a high UPF diet. A high compared with a low UPF diet (62% compared with 42% kcal from UPF) had a slightly lower nutrient composition and quality as reflected by marginally higher levels for nutrients of concern (saturated fat intake, free sugars, sodium), lower intake of fruit, and slightly lower scores on the DHD-CVD index. Additionally, we found some evidence for an association between a high compared with a low UPF diet and stroke mortality risk. We found very limited evidence for an association with overall CVD mortality and no evidence for an association with CHD mortality.

Although significant heterogeneity exists among UPF and not all UPFs are of poor nutritional composition, a diet comprised a majority of these products has consistently demonstrated inferior nutritional quality, displacing whole food. A review conducted across 14 high-, middle-, and low-income countries using nationally representative data demonstrated that UPFs were not eaten infrequently or in isolation but characterized entire dietary patterns and were consumed at the expense of unprocessed food [12], which typically has higher nutritional values. A high compared with a low UPF diet in this review (≥75% kcals compared with ≤15% kcal) was correlated with a substantial increase in free sugars and total and saturated fats, as well as a decrease in fiber, protein, cereals, and fruit [12]. In our study, the differences in UPF intake between extreme quartiles were smaller as was the difference in the nutritional quality as reflected by a composite metric, although fewer cardioprotective whole foods were eaten by the highest UPF consumers.

We found a 21% higher hazard for stroke mortality for every 10% point increase in %g of UPF. A 10 percentage point increase corresponds to the difference between a low UPF diet (mean: ∼9% g/d) compared with a higher UPF diet (mean: ∼19% g/d), reflecting a meaningful shift equivalent to moving across 2 quartiles in our cohort. Hazards for CVD and CHD mortality tended toward a nonsignificant increase. Among other patient samples like patients with colorectal cancer [35] and in people with type 2 diabetes [36], postdiagnostic total UPF intake has been associated with higher CVD-specific mortality. In an Italian cohort with pre-existing CVD, a high UPF diet was associated with an increased hazard of CVD and CHD/cerebrovascular mortality, possibly through mechanisms that include altered renal function through high cystatin C concentrations [37]. The evidence for CVD-specific mortality among nonpatient samples is also inconsistent [38]. Rico-Campà et al. [39] found a trend toward but no statistical significance for increased CVD mortality with high compared with low UPF intake in their Spanish cohort of university graduates. Similar results were seen for cardiometabolic mortality in 2 other United States-based samples [40,41], and for CVD mortality in a United Kingdom sample [42]. Conversely, a higher risk of death from CVD and heart disease but not cerebrovascular disease was found in a population-based United States cohort [43] while another found higher mortality for stroke but not CHD [44].

The mixed findings for stroke, CHD, and CVD mortality warrant further consideration. We observed a direct association with risk of stroke mortality consistent with our hypothesis, but the associations with overall CVD mortality were inconsistent, and no link was found with CHD mortality, which contradicts our hypothesis. Several factors may explain the neutral association between UPF intake and CHD mortality and the adverse association between UPF intake and risk of stroke mortality in our sample. Dietary variation was limited in this older, predominantly White Dutch cohort, and several UPF categories relevant to CVD risk (e.g., sugar-sweetened beverages and processed meat) were consumed in relatively low amounts. Limited exposure contrast may have reduced the power to detect modest associations, particularly for CHD mortality. All participants had a history of MI and thus a high baseline risk of recurrent and fatal CHD (mortality rate: 12/1000 person-years), which may have reduced the ability to detect small relative differences in risk between UPF exposure groups. Therefore, limited exposure contrast and high baseline CVD risk may have reduced power to detect modest associations for total CVD and CHD mortality in our sample.

For stroke mortality risk, we did find an adverse association with UPF intake. The baseline risk of fatal stroke in our cohort was relatively low (mortality rate: 4/1000 person-years) compared with fatal CHD, making elevated relative risks easier to detect. Differences in disease pathways may also have played a role. Although CHD and stroke share atherosclerotic mechanisms, stroke, particularly hemorrhagic stroke, may be more strongly influenced by elevated blood pressure and small vessel damage. Mean systolic blood pressure in our cohort was elevated (141–143 mm Hg across quartiles). If blood pressure control was suboptimal in this population, stroke risk may have been more susceptible to dietary influences. The nutritional profile of a diet high in UPFs, characterized by higher sodium and lower intakes of fruit, potassium, and polyphenols, may contribute to adverse blood pressure pattern and could partly explain the observed associations with fatal stroke.

Finally, as our study focused exclusively on fatal events, differences in case fatality may have influenced the observed associations. Patients with pprior MI typically receive regular clinical follow-up, which may reduce the risk of fatal CHD through timely detection and treatment. In contrast, stroke—particularly when sudden or severe—may occur with less opportunity for medical intervention. Where clinical response is limited, the potential for prevention through diet may become more relevant. Moreover, fatal compared with nonfatal stroke may have accounted for a larger share of total stroke events than was the case for CHD, making fatal stroke a closer proxy for overall stroke risk. For CHD, fatal events captured only part of the burden, and incidence would have provided a more complete picture. Unfortunately, we did not collect long-term follow-up data on nonfatal CVD events in the AOC.

Associations between a high UPF diet and higher mortality risk were stronger among patients who were older, females, with higher BMIs, lower SES, lower activity levels, and former smokers, but also among groups with higher diet quality scores on the composite metric. These results underscore the need for future research to better understand and address the economic barriers that may be faced by patients in meeting CVD guidelines and on processing-specific mechanisms underpinning a high UPF diet. The stronger associations observed among patients with higher diet quality scores may reflect that conventional diet quality indices capture favorable nutrient and food group patterns but may not fully capture potentially adverse aspects of food processing, such as higher sodium content, additives, and alterations in food structure. The consumption of more UPF among participants with higher diet quality potentially contributed to stroke risk, even in the context of otherwise favorable nutrient patterns.

Although our cohort included a substantial group of female MI survivors (*n =* 933), the overall associations between UPF intake and fatal CVD outcomes largely pertain to male participants, who comprised 79% of the cohort. Sex distribution showed a gradient across the lower UPF quartiles, and we therefore assessed potential effect modification by sex through stratified analyses. These analyses suggested a stronger association between UPF intake and stroke risk among females than males; however, formal interaction tests did not support a statistically significant effect modification. Therefore, these findings should be interpreted with caution given the smaller female subcohort.

Potential biological underpinnings of the associations seen in our study are likely to be via a combination of pathways that include: *1*) displacement of minimally processed food by UPF. Our results lend some support to this pathway; *2*) inferior diet quality. Indeed, our association strength was attenuated when the analyses were adjusted for diet quality; and *3*) excess energy intake; associations were strengthened in analyses that controlled for non-UPF subgroups but not total energy. Other hypothesized processing-specific pathways that we were unable to assess in our study include uncovering the role that cosmetic additives, phthalates, and other neoformed compounds may play.

The results of this study should be considered in light of some limitations. Dietary intake was assessed only at baseline, which may result in nondifferential bias. Although UPF intake was assessed only at baseline, dietary assessment was performed on average 3.7 y after the index MI, when participants were likely to have resumed their habitual dietary patterns. Nevertheless, dietary changes during the 14-y follow-up cannot be excluded and may have resulted in nondifferential exposure misclassification, likely attenuating the observed associations. Also, the FFQ was not validated for UPF intake. This could be another source of underreporting and misclassification of the exposure. Given that both sources of misclassification are likely to have been nondifferential and independent, this would likely result in an attenuation of the results toward the null and would explain the nonsignificant associations found in this study [22]. Future studies with validated UPF measures are needed to confirm our findings.

We lacked a reliable assessment of salt intake because we did not collect 24-h urinary samples and were unable to estimate UPF brand-specific sodium content or discretionary salt use with the FFQ. The potential of residual confounding due to unrecognized or unmeasured confounders in this study exists; however, we attempted to minimize these influences by systematically incorporating a wide range of potential confounders in our statistical models. Misclassification of outcomes may have occurred, which may account for the results seen [45]. The study design was prospective with a long follow-up, and the associations we found were robust to several sensitivity analyses, supporting our conclusions. The generalizability of the observed UPF–CVD associations should be considered in the context of the study population. The association may differ across populations with different underlying CVD risk profiles, health care settings, or dietary patterns. Our findings may not directly apply to younger populations or individuals without established CVD, in whom susceptibility to dietary exposures may differ. The smaller number of female patients resulted in less precise sex-specific estimates and uncertainty about generalizability to females. Further research is also needed to determine whether our findings extend to populations with different ethnic backgrounds and non-European descent.

In conclusions, our study found evidence for an association between a high UPF diet and increased risk of stroke mortality among patients who have previously experienced an MI. The results support current CVD treatment and management recommendations to choose a wide range of fruit, unsalted nuts, whole grains, minimally processed food over UPF, and minimize salt, added sugar, and alcohol intake [[46], [47], [48]]. Associations between UPF and stroke mortality risk were stronger among patients with lower educational attainment, lower physical activity levels, and higher rates of smoking with existing diseases, suggesting the need for providing patient support for overcoming structural barriers in meeting CVD guidelines.

## Author contributions

The authors’ responsibilities were as follows – NK, MGJC: designed the research, wrote the manuscript, and primary responsible for the final content; JMG: granted access to the data; MGJC: analyzed the data; and all authors: interpreted the results, read and approved the final manuscript.

## Declaration of Generative AI and AI-Assisted Technologies in the Writing Process

No AI tool was used.

## Data availability

Data described in the manuscript, codebook, and analytic code will be made available on request pending application and approval.

## Funding

This research was supported by seed funding awarded to NK by the Institute 4 Preventive Health, a strategic initiative of the alliance between Eindhoven University of Technology (TU/e), Wageningen University and Research (WUR), Utrecht University (UU), and University Medical Center Utrecht (UMCU). The funding sources had no role in the study design and conduct, data analysis, or manuscript preparation. The Alpha Omega Cohort was supported by the Netherlands Heart Foundation grant 200T401 and NIH, National Heart, Lung, and Blood Institute and Office of Dietary Supplements grant R01HL076200.

## Conflict of interest

The authors report no conflicts of interest. NK serves/has served as a consultant for UNICEF, PAHO, Bloomberg Philanthropies, and Resolve to Save Lives.
