# Supplementary material for: Ultra-processed Food Intake and Long-Term Risk of Cardiovascular Disease Mortality among Patients with Myocardial Infarction: A Prospective Analysis of the Alpha Omega Cohort
Source: Curr Dev Nutr. 2026 Sep 1;10(10):109527. doi: 10.1016/j.cdnut.2026.109527 (PMC13602709; doi:10.1016/j.cdnut.2026.109527)
Supplement: Supplementary file 1 [file mmc1.docx]

**SUPPLEMENTARY FILES**

**Ultra-processed food intake and long-term risk of cardiovascular disease mortality among myocardial infarction patients: A prospective analysis of the Alpha Omega Cohort**

Maria G. Jacobo Cejudo, et al., 2026

## **Supplementary Figure 1.** Flowchart of the selection of study participants

**Supplementary Table 1.** Final categorization of all food frequency items into Nova groups and subgroups

| **FFQ item** | **Name** | **Nova classification** | **Nova subgroups** |
| --- | --- | --- | --- |
| bf016 | Cooked liver | 1 | Read meat and meat-based dishes |
| bf027 | Eggs | 1 | Milk and eggs |
| bf033 | Full-fat milk | 1 | Milk and eggs |
| bf034 | Semi-skimmed milk | 1 | Milk and eggs |
| bf046 | Semi-skimmed yogurt, custard etc. | 1 | Yogurt |
| bf062 | Regular full-fat milk in coffee | 1 | Milk and eggs |
| bf063 | Regular semi-skimmed milk in coffee | 1 | Milk and eggs |
| bf064 | Unknown coffee milk | 1 | Milk and eggs |
| bf065 | Other coffee milk | 1 | Milk and eggs |
| bf072 | Savoury pie, quiche | 1 | Ready-to-heat/eat meals |
| bf073 | Pancakes | 1 | Rice, pasta, and cereal dishes |
| bf080 | Hotchpot | 1 | Ready-to-heat/eat meals |
| bf082 | Oven fries | 1 | Roots and tubers |
| bf096 | Ready-bought fried fish | 1 | White meat and meat-based dishes |
| bf105 | Liver- and kidney products | 1 | Read meat and meat-based dishes |
| bf115 | Nuts and seeds with the warm meal | 1 | Beans, legumes and nuts |
| bf230 | Porridge | 1 | Rice, pasta, and cereal dishes |
| bf231 | Skimmed milk | 1 | Milk and eggs |
| bf232 | Buttermilk | 1 | Milk and eggs |
| bf233 | Other types of milk | 1 | Milk and eggs |
| bf235 | Full-fat yogurt, custard etc. | 1 | Yogurt |
| bf236 | Skimmed yogurt, custard etc. | 1 | Yogurt |
| bf239 | Coffee with caffeine | 1 | Coffee and tea |
| bf240 | Coffee without caffeine | 1 | Coffee and tea |
| bf241 | Cappuccino | 1 | Coffee and tea |
| bf243 | Black tea | 1 | Coffee and tea |
| bf244 | Green tea | 1 | Coffee and tea |
| bf245 | Herbal tea | 1 | Coffee and tea |
| bf246 | Other types of tea | 1 | Coffee and tea |
| bf253 | Semi-skimmed coffee milk | 1 | Milk and eggs |
| bf254 | Regular skimmed milk in coffee | 1 | Milk and eggs |
| bf259 | Rice | 1 | Rice, pasta, and cereal dishes |
| bf260 | Noodles | 1 | Rice, pasta, and cereal dishes |
| bf261 | Italian pasta dishes | 1 | Rice, pasta, and cereal dishes |
| bf263 | Onion soup | 1 | Ready-to-heat/eat meals |
| bf264 | Tomato soup | 1 | Ready-to-heat/eat meals |
| bf269 | Boiled potatoes | 1 | Roots and tubers |
| bf270 | Mashed potatoes | 1 | Roots and tubers |
| bf271 | Baked potatoes | 1 | Roots and tubers |
| bf272 | Other potatoes | 1 | Roots and tubers |
| bf274 | Fries preparation unknown | 1 | Roots and tubers |
| bf275 | Endive | 1 | Leafy and cruciferous vegetables |
| bf276 | Spinach and purslane | 1 | Leafy and cruciferous vegetables |
| bf277 | Brussels sprouts | 1 | Leafy and cruciferous vegetables |
| bf278 | Cauliflower | 1 | Leafy and cruciferous vegetables |
| bf279 | Broccoli | 1 | Leafy and cruciferous vegetables |
| bf280 | Other cabbage | 1 | Leafy and cruciferous vegetables |
| bf281 | Carrots | 1 | Roots and tubers |
| bf282 | Peas | 1 | Beans, legumes and nuts |
| bf283 | Broad beans | 1 | Beans, legumes and nuts |
| bf284 | Green beans | 1 | Beans, legumes and nuts |
| bf285 | Legumes | 1 | Beans, legumes and nuts |
| bf286 | Leek | 1 | Other vegetables |
| bf287 | Chicory | 1 | Other vegetables |
| bf288 | Kohlrabi | 1 | Leafy and cruciferous vegetables |
| bf289 | Beets | 1 | Roots and tubers |
| bf290 | Mushrooms | 1 | Other vegetables |
| bf291 | Bell pepper | 1 | Other vegetables |
| bf292 | Tomatoes | 1 | Other vegetables |
| bf293 | Onion | 1 | Roots and tubers |
| bf294 | Lettuce and crudités | 1 | Leafy and cruciferous vegetables |
| bf295 | Other kind of vegetables | 1 | Leafy and cruciferous vegetables |
| bf298 | Steak etc. | 1 | Read meat and meat-based dishes |
| bf299 | Blind finch etc. | 1 | White meat and meat-based dishes |
| bf300 | Gammon steak etc. | 1 | Read meat and meat-based dishes |
| bf301 | Pork fillet etc. | 1 | Read meat and meat-based dishes |
| bf303 | Chicken/turkey without skin etc. | 1 | White meat and meat-based dishes |
| bf304 | Chicken with skin etc. | 1 | White meat and meat-based dishes |
| bf305 | Minced meat etc. | 1 | Read meat and meat-based dishes |
| bf306 | Other kind of meat | 1 | Read meat and meat-based dishes |
| bf307 | Shellfish | 1 | White meat and meat-based dishes |
| bf308 | Trout, gurnard etc. | 1 | White meat and meat-based dishes |
| bf309 | Herring | 1 | White meat and meat-based dishes |
| bf310 | Eel, mackerel, salmon | 1 | White meat and meat-based dishes |
| bf311 | Other kind of fish | 1 | White meat and meat-based dishes |
| bf334 | Citrus fruits | 1 | Fruit |
| bf335 | Apples, pears | 1 | Fruit |
| bf336 | Bananas | 1 | Fruit |
| bf338 | Strawberries | 1 | Fruit |
| bf339 | Blueberries | 1 | Fruit |
| bf340 | Cherries | 1 | Fruit |
| bf341 | Grapes | 1 | Fruit |
| bf342 | Peaches, nectarines | 1 | Fruit |
| bf343 | Plums | 1 | Fruit |
| bf344 | Apricots | 1 | Fruit |
| bf345 | Kiwis | 1 | Fruit |
| bf347 | Other fruit | 1 | Fruit |
| bf348 | Orange juice | 1 | Fruit juice |
| bf351 | Grapefruit juice | 1 | Fruit juice |
| bf358 | Tap water or diet soda | 1 | Regular or diet soda, sugary drinks |
| bf052 | Sugar in dairy products | 2 | Sugar |
| bf088 | Vinegar or dressing for crudités | 2 | Vegetable oils |
| bf117 | Cream with the warm meal | 2 | Animal fat |
| bf247 | Sugar in coffee | 2 | Sugar |
| bf248 | Sugar in cappuccino etc. | 2 | Sugar |
| bf249 | Sugar in tea | 2 | Sugar |
| bf296 | Olive oil/vinegar dressing for crudités | 2 | Vegetable oils |
| bf297 | Other oil/vinegar dressing for crudités | 2 | Vegetable oils |
| bf401 | Butter | 2 | Animal fat |
| bf401c | Butter (spreads) | 2 | Animal fat |
| bf401h | Butter (food preparation fats) | 2 | Animal fat |
| bf414 | Other types of oil | 2 | Vegetable oils |
| bf414h | Other types of oil (food preparation fats) | 2 | Vegetable oils |
| bf417 | Lard | 2 | Animal fat |
| bf417c | Lard (spreads) | 2 | Animal fat |
| bf417h | Lard (food preparation fats) | 2 | Animal fat |
| bf420 | Other type of spread | 2 | Vegetable oils |
| bf420c | Other type of spread (spreads) | 2 | Vegetable oils |
| bf423 | Unknown type of fat cooked meal | 2 | Vegetable oils |
| bf423h | Unknown type of fat cooked meal (food preparation fats) | 2 | Vegetable oils |
| bf425 | Olive oil | 2 | Vegetable oils |
| bf425h | Olive oil (food preparation fats) | 2 | Vegetable oils |
| bf426 | Deep fying oil | 2 | Vegetable oils |
| bf426h | Deep fying oil (food preparation fats) | 2 | Vegetable oils |
| bf427 | Butter / low-fat butter | 2 | Animal fat |
| bf427h | Butter / low-fat butter (food preparation fats) | 2 | Animal fat |
| bf011 | Luxury cheese, full fat | 3 | Cheese |
| bf012 | Luxury cheese, reduced fat | 3 | Cheese |
| bf013 | Unknown type of cheese | 3 | Cheese |
| bf115 | Cheese with the warm meal | 3 | Cheese |
| bf155 | Nuts | 3 | Nut and legume-based products |
| bf157 | Cubes of cheese | 3 | Cheese |
| bf160 | Beer | 3 | Fermented alcohol |
| bf161 | Low-alcohol- or alcohol-free beer | 3 | Fermented alcohol |
| bf209 | Multigrain bread | 3 | Processed breads |
| bf214 | Crispbread, whole-wheat rusk | 3 | Processed breads |
| bf215 | Whole-wheat crispbread, rye bread | 3 | Processed breads |
| bf217 | Cheese, 20% fat | 3 | Cheese |
| bf218 | Cheese, 30% fat | 3 | Cheese |
| bf219 | Regular cheese | 3 | Cheese |
| bf220 | Other cheese | 3 | Cheese |
| bf225 | Cornflakes | 3 | Breakfast cereals |
| bf226 | Fibre-rich breakfast products | 3 | Breakfast cereals |
| bf227 | Other breakfast products | 3 | Breakfast cereals |
| bf267 | Soup with legumes | 3 | Ready-to-heat/eat meals |
| bf268 | Other soup | 3 | Ready-to-heat/eat meals |
| bf314 | Soya products: tahoe, tofu | 3 | Nut and legume-based products |
| bf315 | Soya products: Tempeh | 3 | Nut and legume-based products |
| bf318 | Tomato sauce | 3 | Processed dressings |
| bf320 | Mustard | 3 | Processed dressings |
| bf328 | With mustard | 3 | Processed dressings |
| bf337 | Apple sauce | 3 | Processed fruit and vegetable-based juices |
| bf346 | Dried fruit | 3 | Processed fruit and vegetable-based juices |
| bf349 | Apple juice | 3 | Processed fruit and vegetable-based juices |
| bf350 | Grape juice | 3 | Processed fruit and vegetable-based juices |
| bf352 | Tomato juice | 3 | Processed fruit and vegetable-based juices |
| bf353 | Vegetable juice | 3 | Processed fruit and vegetable-based juices |
| bf354 | Other juices | 3 | Processed fruit and vegetable-based juices |
| bf361 | Red wine | 3 | Fermented alcohol |
| bf362 | Rosé wine | 3 | Fermented alcohol |
| bf363 | White wine | 3 | Fermented alcohol |
| bf364 | Sherry, Vermouth, port | 3 | Fermented alcohol |
| bf365 | Other alcoholic beverages | 3 | Fermented alcohol |
| bf003 | Croissants | 4 | Ultra-processed grain-based snacks and desserts |
| bf006 | Gingerbread (loaf) | 4 | Ultra-processed breads |
| bf017 | Liver products | 4 | Ultra-processed meat |
| bf018 | Gammon meat | 4 | Ultra-processed meat |
| bf019 | Luncheon meat | 4 | Ultra-processed meat |
| bf020 | Bacon | 4 | Ultra-processed meat |
| bf021 | Unknown type of meat | 4 | Ultra-processed meat |
| bf022 | Other types of meat | 4 | Ultra-processed meat |
| bf023 | Peanutbutter | 4 | Ultra-processed sauces and spreads |
| bf024 | Chocolate spread | 4 | Chocolate and confectionery |
| bf026 | Sandwich spread | 4 | Ultra-processed sauces and spreads |
| bf030 | Packaged breakfast yogurt | 4 | Ultra-processed milk drinks |
| bf039 | Full-fat chocolate milk | 4 | Ultra-processed milk drinks |
| bf040 | Semi-skimmed chocolate milk, yogurt drink etc. | 4 | Ultra-processed milk drinks |
| bf041 | Skimmed chocolate milk, yogurt drink etc. | 4 | Ultra-processed milk drinks |
| bf050 | Ice cream | 4 | Ultra-processed milk-based desserts |
| bf051 | Popsicle | 4 | Chocolate and confectionery |
| bf053 | Whipped cream | 4 | Ultra-processed milk-based desserts |
| bf058 | Coffee-creamer | 4 | Ultra-processed milk drinks |
| bf061 | Evaporated milk/ coffee milk powder | 4 | Ultra-processed milk drinks |
| bf070 | Pizza | 4 | Ready-to-heat/eat meals |
| bf090 | Low fat mayonnaise etc. for crudités | 4 | Ultra-processed sauces and spreads |
| bf091 | Mayonnaise for crudités | 4 | Ultra-processed sauces and spreads |
| bf092 | Unknown dressing for crudités | 4 | Ultra-processed sauces and spreads |
| bf093 | Other dressing for crudités | 4 | Ultra-processed sauces and spreads |
| bf095 | Ready-bought fried fish | 4 | Savory snacks |
| bf106 | Snackbar products | 4 | Savory snacks |
| bf119 | Satay- or peanut sauce | 4 | Ultra-processed sauces and spreads |
| bf122 | Mayonnaise | 4 | Ultra-processed sauces and spreads |
| bf123 | Low-fat mayonnaise | 4 | Ultra-processed sauces and spreads |
| bf124 | Ketchup etc. | 4 | Ultra-processed sauces and spreads |
| bf130 | Biscuits and cookies | 4 | Ultra-processed grain based snacks and desserts |
| bf131 | Large cookies | 4 | Ultra-processed grain based snacks and desserts |
| bf132 | Food rusk | 4 | Ultra-processed grain based snacks and desserts |
| bf133 | Pie or cake | 4 | Ultra-processed grain based snacks and desserts |
| bf137 | Sweets / candies | 4 | Chocolate and confectionery |
| bf139 | Snack: meat ball etc. | 4 | Ultra-processed meat |
| bf140 | Snackbar products | 4 | Savory snacks |
| bf141 | Snack: mini snacks | 4 | Savory snacks |
| bf142 | Snack: fries | 4 | Savory snacks |
| bf143 | Snack: small springroll | 4 | Savory snacks |
| bf144 | Snack: large springroll | 4 | Savory snacks |
| bf145 | Snack: satay | 4 | Savory snacks |
| bf146 | Snack: sausage roll etc. | 4 | Ready-to-heat/eat meals |
| bf147 | Snack: shawarma sandwich | 4 | Ready-to-heat/eat meals |
| bf148 | With mayonnaise | 4 | Ultra-processed sauces and spreads |
| bf149 | With low-fat mayonnaise | 4 | Ultra-processed sauces and spreads |
| bf150 | With ketchup | 4 | Ultra-processed sauces and spreads |
| bf151 | With peanut sauce | 4 | Ultra-processed sauces and spreads |
| bf154 | Salad (huzarenslaatje) | 4 | Ready-to-heat/eat meals |
| bf156 | Potato chips and pretzels | 4 | Savory snacks |
| bf158 | Sausage | 4 | Ultra-processed meat |
| bf163 | Advocaat | 4 | Distilled alcohol |
| bf164 | Mixed/longdrinks | 4 | Distilled alcohol |
| bf165 | Strong liquor | 4 | Distilled alcohol |
| bf206 | White bread | 4 | Ultra-processed breads |
| bf207 | Brown bread, wheat bread | 4 | Ultra-processed breads |
| bf208 | Whole wheat bread | 4 | Ultra-processed breads |
| bf210 | Other bread | 4 | Ultra-processed breads |
| bf211 | White rolls, raisin rolls | 4 | Ultra-processed breads |
| bf212 | White rusk etc. | 4 | Ultra-processed breads |
| bf213 | Brown rolls and muesli rolls | 4 | Ultra-processed breads |
| bf216 | Other bread substitute | 4 | Ultra-processed breads |
| bf221 | Coconut topping (bread) | 4 | Chocolate and confectionery |
| bf222 | Sweet bread toppings | 4 | Chocolate and confectionery |
| bf224 | Granola | 4 | Breakfast cereals |
| bf229 | Pudding | 4 | Ultra-processed milk based desserts |
| bf234 | Other chocolate milk, yogurt drink | 4 | Ultra-processed milk drinks |
| bf237 | Other yogurt, custard enz | 4 | Ultra-processed milk based desserts |
| bf238 | Other types of ice cream | 4 | Ultra-processed milk based desserts |
| bf265 | Broth without filling | 4 | Ready-to-heat/eat meals |
| bf266 | Broth with filling | 4 | Ready-to-heat/eat meals |
| bf273 | Fried fries | 4 | Savory snacks |
| bf302 | Hamburger etc. | 4 | Ready-to-heat/eat meals |
| bf312 | Quorn | 4 | Ready-to-heat/eat meals |
| bf313 | Vegetarian burger | 4 | Ready-to-heat/eat meals |
| bf316 | Cheese soufflé | 4 | Ready-to-heat/eat meals |
| bf317 | Other meat substitutes | 4 | Ultra-processed meat |
| bf319 | Other warm sauces | 4 | Ultra-processed sauces and spreads |
| bf321 | Other cold sauces | 4 | Ultra-processed sauces and spreads |
| bf322 | Candybars | 4 | Chocolate and confectionery |
| bf323 | Pieces of chocolates etc. | 4 | Chocolate and confectionery |
| bf325 | Dark chocolate | 4 | Chocolate and confectionery |
| bf326 | Milk chocolate | 4 | Chocolate and confectionery |
| bf327 | White chocolate | 4 | Chocolate and confectionery |
| bf329 | With other sauce | 4 | Ultra-processed sauces and spreads |
| bf330 | Toast with fish salad | 4 | Ultra-processed breads |
| bf331 | Toast with fish | 4 | Ultra-processed breads |
| bf332 | Toast without fish | 4 | Ultra-processed breads |
| bf355 | Ice tea | 4 | Regular or diet soda, sugary drinks |
| bf356 | Rosehip syrup | 4 | Chocolate and confectionery |
| bf357 | Coke with caffeine | 4 | Regular or diet soda, sugary drinks |
| bf359 | Other soda | 4 | Regular or diet soda, sugary drinks |
| bf360 | Other drinks | 4 | Regular or diet soda, sugary drinks |
| bf402 | Low-fat butter | 4 | Ultra-processed fats, and oils (for cooking purposes) |
| bf402c | Low-fat butter (spreads) | 4 | Ultra-processed sauces and spreads |
| bf402h | Low-fat butter (food preparation fats) | 4 | Ultra-processed fats, and oils (for cooking purposes) |
| bf403 | Low-fat margarine | 4 | Ultra-processed sauces and spreads |
| bf403c | Low-fat margarine (spreads) | 4 | Ultra-processed sauces and spreads |
| bf404 | Low-fat diet margarine | 4 | Ultra-processed sauces and spreads |
| bf404c | Low-fat diet margarine (spreads) | 4 | Ultra-processed sauces and spreads |
| bf405 | Cholesterol-lowering low-fat margarine | 4 | Ultra-processed sauces and spreads |
| bf405c | Cholesterol-lowering low-fat margarine (spreads) | 4 | Ultra-processed sauces and spreads |
| bf406 | Tub margarine | 4 | Ultra-processed sauces and spreads |
| bf406c | Tub margarine (spreads) | 4 | Ultra-processed sauces and spreads |
| bf406h | Tub margarine (food preparation fats) | 4 | Ultra-processed sauces and spreads |
| bf407 | Stick margarine | 4 | Ultra-processed sauces and spreads |
| bf407c | Stick margarine (spreads) | 4 | Ultra-processed sauces and spreads |
| bf407h | Stick margarine (food preparation fats) | 4 | Ultra-processed fats, and oils (for cooking purposes) |
| bf408 | Diet margarine | 4 | Ultra-processed sauces and spreads |
| bf408c | Diet margarine (spreads) | 4 | Ultra-processed sauces and spreads |
| bf408h | Diet margarine (food preparation fats) | 4 | Ultra-processed fats, and oils (for cooking purposes) |
| bf409 | Cholesterol-lowering margarine | 4 | Ultra-processed fats, and oils (for cooking purposes) |
| bf409c | Cholesterol-lowering margarine (spreads) | 4 | Ultra-processed sauces and spreads |
| bf409h | Cholesterol-lowering margarine (food preparation fats) | 4 | Ultra-processed fats, and oils (for cooking purposes) |
| bf410 | Liquid margarine | 4 | Ultra-processed fats, and oils (for cooking purposes) |
| bf410h | Liquid margarine (food preparation fats) | 4 | Ultra-processed fats, and oils (for cooking purposes) |
| bf411 | Solid bake and frying fat | 4 | Ultra-processed fats, and oils (for cooking purposes) |
| bf411h | Solid bake and frying fat (food preparation fats) | 4 | Ultra-processed fats, and oils (for cooking purposes) |
| bf412 | Liquid bake and frying fat | 4 | Ultra-processed fats, and oils (for cooking purposes) |
| bf412h | Liquid bake and frying fat (food preparation fats) | 4 | Ultra-processed fats, and oils (for cooking purposes) |
| bf415 | Solid deepfrying oil | 4 | Ultra-processed fats, and oils (for cooking purposes) |
| bf415h | Solid deepfrying oil (food preparation fats) | 4 | Ultra-processed fats, and oils (for cooking purposes) |
| bf416 | Liquid deep frying oil | 4 | Ultra-processed fats, and oils (for cooking purposes) |
| bf416h | Liquid deep frying oil (food preparation fats) | 4 | Ultra-processed fats, and oils (for cooking purposes) |
| bf419 | Unknown type of spread | 4 | Ultra-processed sauces and spreads |
| bf419c | Unknown type of spread (spreads) | 4 | Ultra-processed sauces and spreads |
| bf421 | Unknown types of fat cooked vegetables | 4 | Ultra-processed fats, and oils (for cooking purposes) |
| bf421h | Unknown types of fat cooked vegetables (food preparation fats) | 4 | Ultra-processed fats, and oils (for cooking purposes) |
| bf422 | Other types of fat cooked vegetables | 4 | Ultra-processed fats, and oils (for cooking purposes) |
| bf422h | Other types of fat cooked vegetables (food preparation fats) | 4 | Ultra-processed fats, and oils (for cooking purposes) |
| bf424 | Other type of fat cooked meal | 4 | Ultra-processed fats, and oils (for cooking purposes) |
| bf424h | Other type of fat cooked meal (food preparation fats) | 4 | Ultra-processed fats, and oils (for cooking purposes) |

**Supplementary Table 2.** Food items included in the 13 subgroups of ultra-processed foods

| **#** | **Ultra-processed food subgroups** | **Specific food items** |
| --- | --- | --- |
| 1 | Breakfast cereals | Muesli, cruesli |
| 2 | Chocolate and confectionery | Chocolate spread; Popsicle; Sweets / candies; Candy bars; Chocolate |
| 3 | Distilled alcohol [excluded from the main analysis] | Advocaat; Mixed/long drinks; Strong liquor |
| 4 | Ready-to-heat/eat meals | Pizza; Snack: sausage roll etc.; Snack: shawarma sandwich; French fries,  Hamburger etc.; Quorn; Vegetarian burger; Cheese soufflé |
| 5 | Regular or diet soda, sugary drinks | Ice tea; Coke with caffeine |
| 6 | Savory snacks | Snack bar products; Snack: mini snacks; Snack: fries; Potato chips and pretzels |
| 7 | Ultra-processed breads | Gingerbread (loaf); White bread; Brown bread, wheat bread; Other bread; White rolls, raisin rolls; White rusk etc.; Brown rolls and muesli rolls |
| 8 | Ultra-processed fats and oils for cooking | Low-fat butter (food preparation fats); Stick margarine (food preparation fats); Diet margarine (food preparation fats); Cholesterol-lowering margarine |
| 9 | Ultra-processed grain-based snacks | Croissants; Biscuits and cookies; Large cookies; Food rusk; Pie or cake |
| 10 | Ultra-processed meats | Gammon meat; Luncheon meat; Bacon; Unknown type of meat; Other types of meat; Sausage; Other meat substitutes |
| 11 | Ultra-processed dairy-based desserts | Ice cream; Whipped cream; Pudding; Other yogurt, custard enz |
| 12 | Ultra-processed dairy-based drinks | Packaged breakfast yogurt; Full-fat chocolate milk; Semi-skimmed chocolate milk, yogurt drink etc.; Skimmed chocolate milk, yogurt drink etc.; Other chocolate milk, yogurt drink |
| 13 | Ultra-processed sauces and spreads | Peanut butter; Sandwich spread; Low fat mayonnaise etc. for crudités;  Satay- or peanut sauce; Ketchup etc. |

## **Supplementary Table 3.** Hazard ratios (95% CIs) of the association between ultra-processed food (UPF) consumption and risk of mortality from cardiovascular disease (CVD), ischemic heart disease (IHD) and stroke, among 4,365 post-myocardial infarction patients of the Alpha Omega Cohort, across different sensitivity analysis

|  | **UPF quartiles**  **% g/day^a^** | | | | **Per 10% point increment in the % UPF g/day^b^** | **Per 1 SD increment in energy adjusted UPF intake**  **(169 g/day)^c^** |
| --- | --- | --- | --- | --- | --- | --- |
|  | Q1  ≤12% | Q2  >12-≤16% | Q3  >16-≤22% | Q4  >22% |  |  |
| Total CVD mortality |  |  |  |  |  |  |
| Non-cases/cases | 811/281 | 816/275 | 813/277 | 813/279 | 3,253/1,112 | 3,253/1,112 |
| Main model | Ref. | 0.99 (0.83, 1.17) | 0.99 (0.83, 1.17) | 1.04 (0.87,1.24) | 1.04 (0.96, 1.12) | 1.06 (1.00, 1.13) |
| Model S1 | Ref. | 1.00 (0.84, 1.18) | 1.00 (0.84, 1.18) | 1.03 (0.87, 1.23) | 1.05 (0.97, 1.13) | 1.07 (1.01, 1.13)* |
| Model S2 | Ref. | 0.97 (0.82, 1.15) | 0.98 (0.83, 1.17) | 1.03 (0.86, 1.22) | 1.04 (0.96, 1.12) | 1.06 (1.00, 1.13) |
| Model S3 | Ref. | 0.96 (0.81, 1.14) | 0.94 (0.79, 1.12) | 0.95 (0.79, 1.14) | 1.00 (0.92, 1.08) | 1.04 (0.98, 1.11) |
| Model S4^d^ | Ref. | 0.96 (0.82, 1.13) | 0.82 (0.69, 0.97)* | 0.85 (0.71, 1.01) | 0.95 (0.90, 1.00) | NA |
| Model S5 | Ref. | 1.00 (0.83, 1.19) | 1.01 (0.84, 1.22) | 1.11 (0.92, 1.34) | 1.06 (0.98, 1.15) | 1.07 (1.00, 1.14) |
| Model S6 | Ref. | 1.00 (0.85, 1.19) | 1.01 (0.85, 1.20) | 1.07 (0.90, 1.28) | 1.06 (0.98, 1.14) | 1.09 (1.02, 1.16)* |
| Model S7 | Ref. | 0.98 (0.83, 1.16) | 0.98 (0.82, 1.17) | 1.03 (0.86, 1.23) | 1.04 (0.96, 1.12) | 1.06 (1.00, 1.13) |
| Model S8 | Ref. | 0.99 (0.83, 1.17) | 0.99 (0.83, 1.17) | 1.04 (0.87, 1.24) | 1.04 (0.96, 1.12) | 1.06 (1.00, 1.13) |
| Total IHD mortality |  |  |  |  |  |  |
| Non-cases/cases | 915/177 | 928/163 | 937/153 | 926/166 | 3,706/659 | 3,706/659 |
| Main model | Ref. | 0.92 (0.74, 1.14) | 0.84 (0.67, 1.05) | 0.94 (0.75, 1.18) | 1.01 (0.91, 1.12) | 1.05 (0.97, 1.14) |
| Model S1 | Ref. | 0.91 (0.73, 1.13) | 0.89 (0.72, 1.11) | 0.94 (0.75, 1.18) | 1.02 (0.92, 1.12) | 1.05 (0.97, 1.14) |
| Model S2 | Ref. | 0.90 (0.73, 1.12) | 0.84 (0.67, 1.05) | 0.93 (0.74, 1.17) | 1.01 (0.91, 1.11) | 1.04 (0.97, 1.13) |
| Model S3 | Ref. | 0.90 (0.72, 1.11) | 0.80 (0.64, 1.01) | 0.87 (0.69, 1.10) | 0.97 (0.87, 1.08) | 1.03 (0.95, 1.12) |
| Model S4^d^ | Ref. | 0.88 (0.71, 1.09) | 0.77 (0.62, 0.96)* | 0.76 (0.61, 0.95)* | 0.91 (0.85, 0.98)* | NA |
| Model S5 | Ref. | 0.91 (0.71, 1.15) | 0.86 (0.67, 1.10) | 1.04 (0.82, 1.33) | 1.05 (0.95, 1.17) | 1.07 (0.98, 1.17) |
| Model S6 | Ref. | 0.92 (0.74, 1.15) | 0.84 (0.67, 1.06) | 0.95 (0.76, 1.20) | 1.02 (0.92, 1.13) | 1.07 (0.99, 1.16) |
| Model S7 | Ref. | 0.92 (0.74, 1.14) | 0.84 (0.67, 1.05) | 0.94 (0.75, 1.18) | 1.01 (0.91, 1.12) | 1.05 (0.97, 1.14) |
| Model S8 | Ref. | 0.92 (0.74, 1.14) | 0.84 (0.67, 1.05) | 0.94 (0.75, 1.18) | 1.01 (0.91, 1.12) | 1.04 (0.96, 1.13) |
| Total stroke mortality |  |  |  |  |  |  |
| Non-cases/cases | 1,046/ 46 | 1,046/45 | 1,032/58 | 1,033/59 | 4,157/208 | 4,157/208 |
| Main model | Ref. | 0.98 (0.64, 1.48) | 1.37 (0.91, 2.06) | 1.47 (0.98, 2.22) | 1.21 (1.02, 1.42)* | 1.21 (1.08, 1.37)* |
| Model S1 | Ref. | 0.92 (0.60, 1.41) | 1.26 (0.84, 1.87) | 1.51 (1.02, 2.26)* | 1.22 (1.03 1.43)* | 1.22 (1.08, 1.37)* |
| Model S2 | Ref. | 0.97 (0.64, 1.48) | 1.38 (0.92, 2.07) | 1.47 (0.98, 2.22) | 1.21 (1.02 1.42)* | 1.21 (1.08, 1.37)* |
| Model S3 | Ref. | 0.95 (0.62, 1.44) | 1.29 (0.86, 1.95) | 1.32 (0.86, 2.03) | 1.15 (0.96 1.37) | 1.18 (1.04, 1.34)* |
| Model S4^d^ | Ref. | 1.01 (0.68, 1.49) | 0.92 (0.62, 1.38) | 1.14 (0.76, 1.70) | 1.06 (0.93, 1.20) | NA |
| Model S5 | Ref. | 1.11 (0.72, 1.71) | 1.42 (0.92, 2.18) | 1.57 (1.02, 2.42)* | 1.20 (1.01 1.43)* | 1.19 (1.05, 1.36)* |
| Model S6 | Ref. | 0.98 (0.64, 1.49) | 1.37 (0.92, 2.05) | 1.48 (0.98, 2.24) | 1.22 (1.03 1.44)* | 1.23 (1.08, 1.39)* |
| Model S7 | Ref. | 0.96 (0.63, 1.46) | 1.34 (0.89, 2.01) | 1.42 (0.94, 2.16) | 1.19 (1.01 1.41)* | 1.20 (1.06, 1.36)* |
| Model S8 | Ref. | 0.98 (0.65, 1.49) | 1.37 (0.91, 2.05) | 1.47 (0.98, 2.21) | 1.20 (1.02, 1.42)* | 1.21 (1.07, 1.37)* |

UPF consumption was operationalized excluding alcohol intake (g/day). ^a^HR and 95% CI across quintiles of the proportion UPF in g/day; ^b^per 10% point increment in the proportion UPF g/day; ^c^per 1 SD increment in energy-adjusted UPF intake in g/day, using the residual method. Standardized residuals were computed by a linear regression of baseline ultra-processed food intake (g/day) regressed on energy intake. All estimated from Cox proportional hazard models. Main model was model 3 adjusted for sex (female/male), educational attainment (only elementary, low, moderate, and high), SES-WOA (score), smoking (never, former, and current), physical activity (no or light activity, intermediate, and high), total ethanol intake (no or very light, light, moderate, and high), and total energy intake (kcal/day).

Model S1 main model using sex-specific UPF quartiles (% g/day)

Model S2 main model additionally adjusted for BMI

Model S3 main model additionally adjusted for diet quality score modified for CVD patients. The Dutch healthy diet index assessed adherence to the Dutch dietary guidelines for CVD patients.

Model S4 main model using UPF consumption in % kcal/day instead of % g/day. Quartiles of kcal/day were as follows: Q1= ≤45%; Q2= >45-≤54%; Q3= >54-≤61%; and Q4= >61%. ^d^per 10% increment in the % kcal/day.

Model S5 main model after excluding the first three years of follow-up

Model S6 main model adjusted for energy intake coming from Nova Groups 1, 2 and 3 foods instead of total energy

Model S7 main model additionally adjusted for sodium intake from foods

Model S8 main model using multiple imputation for missing data in covariates instead of age and gender-specific median/mode.

## **Supplementary Table 4.** Hazard ratios (95% CIs) of the association between ultra-processed food (UPF) consumption and risk of mortality from cardiovascular disease among subgroups of age, sex, BMI, diet quality, physical activity level, being assigned to the intervention arm, social economic status and smoking status^1^

|  |  | **Per 10% point increment in the % UPF g/day** | **Per 1 SD increment in energy adjusted UPF intake in g/day^2^** |
| --- | --- | --- | --- |
|  | Cases/non-cases | HR (95% CI) | HR (95% CI) |
| Age |  |  |  |
| Younger (≤69 years old) | 377/1,805 | 0.99 (0.87, 1.12) | 0.99 (0.89, 1.10) |
| Older (>69 years old) | 735/1,448 | 1.07 (0.98, 1.18) | 1.11 (1.03, 1.20)* |
| Sex |  |  |  |
| Men | 841/2,591 | 1.03 (0.94, 1.12) | 1.05 (0.98, 1.13) |
| Women | 271/662 | 1.11 (0.94, 1.32) | 1.12 (0.99, 1.26) |
| BMI |  |  |  |
| <30kg/m^2^ | 810/2,523 | 1.05 (0.96, 1.15) | 1.06 (0.99, 1.14) |
| ≥30kg/m^2^ | 302/730 | 1.00 (0.86, 1.16) | 1.06 (0.94, 1.20) |
| Diet quality |  |  |  |
| Lower diet quality score (≤88) | 595/1,587 | 0.96 (0.87, 1.06) | 0.99 (0.91, 1.08) |
| Higher diet quality score (>88) | 517/1,666 | 1.09 (0.95, 1.25) | 1.12 (1.03, 1.22)* |
| Physical activity |  |  |  |
| No activity-light | 555/1,237 | 1.02 (0.92, 1.14) | 1.10 (1.01, 1.19)* |
| Moderate-vigorous | 557/2,016 | 1.06 (0.95, 1.19) | 1.04 (0.95, 1.13) |
| Intervention group |  |  |  |
| Intervention | 835/2,423 | 1.05 (0.96, 1.15) | 1.08 (1.01, 1.15)* |
| Placebo | 277/830 | 1.00 (0.86, 1.17) | 1.02 (0.90, 1.16) |
| Social economic status |  |  |  |
| Lower SES-WOA score | 481/1,533 | 1.13 (1.01, 1.26)* | 1.14 (1.04, 1.24)* |
| Higher SES-WOA score | 631/1,720 | 0.97 (0.87, 1.07) | 1.01 (0.93, 1.10) |
| Smoking |  |  |  |
| Never smokers | 187/535 | 1.18 (0.97, 1.43) | 1.15 (1.00, 1.32) |
| Former smokers | 732/2,198 | 1.04 (0.94, 1.14) | 1.06 (0.98, 1.14) |
| Current smokers | 193/520 | 0.95 (0.79, 1.14) | 1.02 (0.88, 1.18) |

^1^Multivariable models were adjusted for sex (except when stratified by sex), educational attainment (except for when stratified by education), SES-WOA (except for when stratified by SES-WOA score), smoking (except for when stratified by smoking status), physical activity (except when stratified by physical activity level), total ethanol intake (categories), and total energy intake (kcal/day). ^2^Using the residual method.

## **Supplementary Table 5.** Hazard ratios (95% CIs) of the association between ultra-processed food (UPF) consumption and risk of mortality from ischemic heart disease among subgroups of age, sex, BMI, diet quality, physical activity level, being assigned to the intervention arm, social economic status and smoking status^1^

|  |  | **Per 10% point increment**  **in the % UPF g/day** | **Per 1 SD increment in energy adjusted UPF intake in g/day^2^** |
| --- | --- | --- | --- |
|  | Cases/non-cases | HR (95% CI) | HR (95% CI) |
| Age |  |  |  |
| Younger (≤69 years old) | 224/1,958 | 0.96 (0.81, 1.15) | 0.99 (0.86, 1.14) |
| Older (>69 years old) | 435/1,748 | 1.04 (0.92, 1.18) | 1.09 (0.99, 1.20) |
| Sex |  |  |  |
| Men | 517/2,915 | 1.00 (0.89, 1.11) | 1.02 (0.93, 1.12) |
| Women | 142/ 791 | 1.11 (0.88, 1.40) | 1.15 (0.99, 1.34) |
| BMI |  |  |  |
| <30kg/m^2^ | 483/2,850 | 1.06 (0.95, 1.19) | 1.05 (0.96, 1.15) |
| ≥30kg/m^2^ | 176/856 | 0.87 (0.71, 1.07) | 1.04 (0.88, 1.22) |
| Diet quality |  |  |  |
| Lower diet quality score (≤88) | 346/1,836 | 0.90 (0.79, 1.03) | 0.96 (0.85, 1.08) |
| Higher diet quality score (>88) | 313/1,870 | 1.16 (0.98, 1.37) | 1.14 (1.02, 1.26)* |
| Physical activity |  |  |  |
| No activity-light | 335/1,457 | 0.99 (0.86, 1.14) | 1.07 (0.96, 1.20) |
| Moderate-vigorous | 324/2,249 | 1.03 (0.89, 1.20) | 1.03 (0.92, 1.15) |
| Intervention group |  |  |  |
| Intervention | 494/2,764 | 1.04 (0.93, 1.1) | 1.07 (0.98, 1.17) |
| Placebo | 165/942 | 0.93 (0.75, 1.14) | 0.97 (0.82, 1.15) |
| Social economic status |  |  |  |
| Lower SES-WOA score | 277/1,737 | 1.10 (0.94, 1.27) | 1.11 (0.99, 1.26) |
| Higher SES-WOA score | 382/1,969 | 0.95 (0.83, 1.09) | 1.01 (0.91, 1.13) |
| Smoking |  |  |  |
| Never smokers | 102/620 | 1.11 (0.85, 1.45) | 1.14 (0.93, 1.40) |
| Former smokers | 439/2,491 | 1.03 (0.91, 1.16) | 1.06 (0.96, 1.17) |
| Current smokers | 118/595 | 0.88 (0.69, 1.12) | 0.98 (0.81, 1.20) |

^1^Multivariable models were adjusted for sex (except when stratified by sex), educational attainment (except for when stratified by education), SES-WOA (except for when stratified by SES-WOA score), smoking (except for when stratified by smoking status), physical activity (except when stratified by physical activity level), total ethanol intake (categories), and total energy intake (kcal/day). ^2^Using the residual method.

## **Supplementary Table 6.** Hazard ratios (95% CIs) of the association between ultra-processed food (UPF) consumption and risk of mortality from stroke among subgroups of age, sex, BMI, diet quality, physical activity level, being assigned to the intervention arm, social economic status and smoking status^1^

|  |  | **Per 10% point increment in the % UPF g/day** | **Per 1 SD increment in energy adjusted UPF intake in g/day^2^** |
| --- | --- | --- | --- |
|  | Cases/non-cases | HR (95% CI) | HR (95% CI) |
| Age |  |  |  |
| Younger (≤69 years old) | 76/2,106 | 1.08 (0.81, 1.43) | 1.09 (0.88, 1.34) |
| Older (>69 years old) | 132/2,051 | 1.31 (1.07, 1.61)* | 1.31 (1.13, 1.52)* |
| Sex |  |  |  |
| Men | 154/3,278 | 1.16 (0.96, 1.40) | 1.16 (1.00, 1.34) |
| Women | 54/879 | 1.38 (0.98, 1.94) | 1.33 (1.08, 1.62)* |
| BMI |  |  |  |
| <30kg/m^2^ | 162/3,171 | 1.13 (0.94, 1.38) | 1.15 (1.00, 1.32) |
| ≥30kg/m^2^ | 46/986 | 1.41 (1.03, 1.94)* | 1.39 (1.10, 1.77)* |
| Diet quality |  |  |  |
| Lower diet quality score (≤88) | 121/2,061 | 1.02 (0.82, 1.25) | 1.02 (0.85, 1.22) |
| Higher diet quality score (>88) | 87/2,096 | 1.33 (0.98, 1.81) | 1.34 (1.14, 1.59)* |
| Physical activity |  |  |  |
| No activity-light | 95/1,697 | 1.22 (0.98, 1.53) | 1.33 (1.13, 1.57)* |
| Moderate-vigorous | 113/2,460 | 1.19 (0.94, 1.50) | 1.11 (0.93, 1.33) |
| Intervention group |  |  |  |
| Intervention | 159/ 3,099 | 1.27 (1.06, 1.53)* | 1.25 (1.10, 1.43)* |
| Placebo | 49/1,058 | 1.00 (0.69, 1.44) | 1.05 (0.79, 1.41) |
| Social economic status |  |  |  |
| Low SES-WOA score | 84/1,930 | 1.26 (0.99, 1.60) | 1.25 (1.04, 1.51)* |
| High SES-WOA score | 124/2,227 | 1.14 (0.92, 1.43) | 1.18 (1.00, 1.38) |
| Smoking |  |  |  |
| Never smokers | 40/682 | 1.41 (0.96, 2.07) | 1.21 (0.91, 1.62) |
| Former smokers | 129/2,801 | 1.14 (0.92, 1.42) | 1.24 (1.07, 1.44)* |
| Current smokers | 39/674 | 1.21 (0.87, 1.67) | 1.14 (0.84, 1.53) |

^1^Multivariable models were adjusted for sex (except when stratified by sex), educational attainment (except for when stratified by education), SES-WOA (except for when stratified by SES-WOA score), smoking (except for when stratified by smoking status), physical activity (except when stratified by physical activity level), total ethanol intake (categories), and total energy intake (kcal/day). ^2^Using the residual method.

**Supplementary Table 7.** Hazard ratios (95% CIs) between unprocessed and minimally processed food consumption (Nova 1) and risk of mortality from cardiovascular disease (CVD), ischemic heart disease (IHD) and stroke, among 4,365 post-myocardial infarction patients of the Alpha Omega Cohort

|  | **Unprocessed/minimally processed food intake, quartiles % g/day** | | | | **Per 10% point increment in the % g/day** |
| --- | --- | --- | --- | --- | --- |
|  | Q1  ≤70% | Q2  >70-≤77% | Q3  >77-≤82% | Q4  >82% |  |
| Total CVD mortality | |  |  |  |  |
| Non-cases /cases | 787/304 | 829/263 | 814/277 | 823/268 | 3,253/1,112 |
| Model 3 | Ref. | 0.84 (0.71, 0.99)* | 0.85 (0.72, 1.00) | 0.81 (0.68, 0.97)* | 0.92 (0.86, 0.99)* |
| Total IHD mortality |  |  |  |  |  |
| Non-cases /cases | 911/180 | 935/157 | 931/160 | 929/162 | 3,706/659 |
| Model 3 | Ref. | 0.85 (0.68, 1.05) | 0.84 (0.68, 1.05) | 0.86 (0.68, 1.07) | 0.93 (0.85, 1.01) |
| Total stroke mortality | |  |  |  |  |
| Non-cases /cases | 1,024/67 | 1,042/ 50 | 1,045/46 | 1,046/45 | 4,157/208 |
| Model 3 | Ref. | 0.70 (0.48, 1.01) | 0.62 (0.42, 0.92)* | 0.60 (0.40, 0.90)* | 0.81 (0.70, 0.93)* |

^1^Model 3 was adjusted for sex, educational level (categories), SES-WOA (continuous), smoking (categories), physical activity (categories), total ethanol intake (categories), and total energy intake (kcal/day).
